# Cutaneous lupus erythematosus: a review of etiopathogenic, clinical, diagnostic and therapeutic aspects^☆^

**DOI:** 10.1016/j.abd.2022.09.005

**Published:** 2023-03-01

**Authors:** Everton Carlos Siviero do Vale, Lucas Campos Garcia

**Affiliations:** a Dermatology Service, Hospital das Clínicas, Universidade Federal de Minas Gerais, Belo Horizonte, MG, Brazil; b Department of Internal Medicine, Faculty of Medicine, Universidade Federal de Minas Gerais, Belo Horizonte, MG, Brazil

**Keywords:** Autoimmune diseases, Lupus erythematosus, cutaneous, Lupus erythematosus, discoid, Lupus erythematosus, systemic, Panniculitis, lupus erythematosus

## Abstract

Cutaneous lupus erythematosus is an autoimmune disease of varied clinical expression, which may present as an exclusively cutaneous disease or be one of the multiple manifestations of systemic lupus erythematosus. Its classification includes acute, subacute, intermittent, chronic and bullous subtypes, which are usually identified based on clinical features and histopathological and laboratory findings. Other non-specific cutaneous manifestations may be associated with systemic lupus erythematosus and are usually related to disease activity. Environmental, genetic and immunological factors play a role in the pathogenesis of skin lesions in lupus erythematosus. Recently, considerable progress has been made in elucidating the mechanisms involved in their development, which allows for foreseeing future targets for more effective treatments. This review proposes to discuss the main etiopathogenic, clinical, diagnostic and therapeutic aspects of cutaneous lupus erythematosus, aiming to update internists and specialists from different areas.

## Introduction

Lupus erythematosus (LE) is an autoimmune disease with a wide spectrum of clinical expression, ranging from limited cutaneous disease to severe and life-threatening systemic disease due to vital-organ involvement.^1^ Cutaneous LE (CLE) presents as an exclusive cutaneous disease or comprises one of the multiple manifestations of systemic LE (SLE). Skin lesions are present in 70%‒80% of SLE cases at some point during their course and may be the initial disease manifestation in up to 25% of patients.^1^

Based on the clinical features, histopathological findings, laboratory findings and duration, LE-specific skin lesions are subdivided into three main subtypes – acute CLE (ACLE), subacute CLE (SCLE) and chronic CLE (CCLE). The identification of these subtypes is crucial, as they often occur in different clinical contexts, with diagnostic, prognostic, and therapeutic implications.^2^

Exclusive CLE is two to three times more frequent than SLE, with an annual incidence of 4.3/100,000 in Europe and the US.^1^, ^2^, ^3^ There is a predominance of LE in the female sex, where the incidence of adult SLE is 7 to 15 times higher and, for childhood SLE, 3 to 4 times higher. This female predominance is less evident in the isolated cutaneous forms of LE, with a ratio of 4:1, and it is even less significant, with a ratio of 3:1, for discoid LE (DLE), the most common form of CCLE.^2^, ^4^

There are also racial differences in the occurrence of CLE, with a 5.4-fold higher risk of CCLE in African-Americans when compared to Caucasians.^4^ In New Zealand, when compared to the population of European origin, the indigenous Māori population shows a relative risk of 2.47 for the development of all CLE subtypes and 5.96 risks for CCLE.^5^

The peak incidence of SLE occurs in middle age, but it occurs later in men.^6^ Although it also affects children and the elderly, exclusive CLE is more common between the ages of 20 and 40 years, with a mean age at onset of 43 years, varying according to the subtype.^3^

LE skin lesions cause considerable morbidity, mainly due to their chronic nature, the preferential involvement of exposed parts of the body, and the disfiguring characteristics of their sequelae, which result in significant patient quality of life impairment.^7^

## Classification

The diagnostic criteria for classifying SLE are not uniform or universally accepted, with those proposed in 1971 by the American College of Rheumatology (ACR) being the precursors, revised in 1997 (ACR 1997); subsequently, two additional classification systems emerged – that of the Systemic Lupus International Collaborating Clinics (SLICC 2012) and the joint one between the European League Against Rheumatism and the ACR (EULAR/ACR 2019), which are differentiated and can be compared in Table 1.^4^, ^8^, ^9^ Each of the three systems above includes four dermatological findings as diagnostic criteria for SLE. A current Australian study, which evaluated the performance of different SLE classifications, concluded that the ACR 1997 criteria showed the highest specificity; however, the SLICC 2012 provided the highest overall diagnostic accuracy (94.4%), with similar performance between patients with early disease.^10^

**Table 1:** Classification criteria for systemic lupus erythematosus – ACR 1997, SLICC 2012 and EULAR/ACR 2019.

ACR 1997^4^	SLICC 2012^8^	EULAR/ACR 2019^9^
**Entry criteria**: none	**Entry criteria**: none	**Entry criteria**: ANA ≥ 1:80
*Clinical criteria (=9)*	*Clinical criteria (=11)*	*Clinical criteria (score)*
**Malar rash**	**Acute, subacute CLE or bullous LE**	**Constitutional**
**Discoid rash**	**Chronic CLE (includes tumid CLE)**	Fever (2)
**Photosensitivity**	**Oral or nasal ulcers**	**Hematological**
**Oral ulcers**	**Non-cicatricial alopecia**	Leukopenia (3)
**Non-erosive arthritis (≥2 joints)**	**Synovitis (≥2 joints)**	Thrombocytopenia (4)
**Serositis**	**Serositis**	Autoimmune hemolysis (4)
Pleuritis	Pleuritis	**Neuropsychiatric**
Pericarditis	Pericarditis	Delirium (2)
Pleural or pericardial effusion	Pleural or pericardial effusion	Psychosis (3)
**Renal involvement**	**Kidney disease**	Seizure (5)
Proteinuria > 0.5 g/24 h	Proteinuria > 0.5 g/24 h	**Mucocutaneous**
Cell casts	Hematic casts	Non-cicatricial alopecia (2)
**Neurological alterations**	**Neurological disease**	Oral ulcers (2)
Seizures	Seizures	Subacute CLE or discoid LE (4)
Psychosis	Psychosis	Acute CLE (6)
**Hematological alterations**	Mononeuritis multiplex	**Serositis**
Hemolytic anemia	Myelitis	Pleural or pericardial effusion (5)
Leukopenia (<4000/mm^3^)	Cranial or peripheral neuropathy	Acute pericarditis (6)
Lymphopenia (<1500/mm^3^)	Acute confusional state	**Musculoskeletal**
Thrombocytopenia (<100,000/mm^3^)	Hemolytic anemia	Joint involvement (6)
	Leukopenia (<4,000/ mm^3^) or lymphopenia (<1,000/mm^3^)	**Renal**
	Thrombocytopenia (<100,000/mm^3^)	Proteinuria > 0.5 g/24 h (4)
		Kidney biopsy – lupus nephritis
		Class II or V (8)
		Kidney biopsy – lupus nephritis
		Class III or IV (10)
*Immunological criteria (=2)*	*Immunological criteria (=6)*	*Immunological criteria (score)*
ANA	ANA	Antiphospholipid antibodies
Autoantibodies	Anti-native DNA	Anticardiolipin (2)
Anti-native DNA	Anti-Sm	Anti-β2-glycoprotein 1 (2)
Anti-Sm	Antiphospholipid antibody	Lupus anticoagulant (2)
Antiphospholipid antibody	Lupus anticoagulant	Complement
	False-positive non-treponemal test	Low C3 or C4 (3)
	Anticardiolipin	Low C3 and C4 (4)
	Anti-β2-glycoprotein 1	LES-specific antibodies
	Low complement (C3, C4 or CH50)	Anti-native DNA (6)
	Direct Coombs (in the absence of hemolytic anemia)	Anti-Sm (6)

ACR, American College of Rheumatology; EULAR, European League Against Rheumatism; SLICC, Systemic Lupus International Collaborating Clinics; ANA, Antinuclear Antibody; CLE, Cutaneous Lupus Erythematosus; SLE, Systemic Lupus Erythematosus.

ACR 1997: SLE = ≥4 criteria.

SLICC 2012: SLE = ≥4 criteria, at least one clinical and one immunological; biopsy-proven lupus nephritis with ANA or anti-native DNA can also be classified as SLE.

EULAR/ACR 2019: SLE = score ≥ 10 points, at least one clinical (only the criterion with the highest score in each domain is considered).

The dermatologic criteria are highlighted in bold in all three classification systems.

The classification proposed by Gilliam & Sontheimer, in 1981, was pioneer and differentiates LE cutaneous lesions in specific and nonspecific ones. The specific ones, defined by the presence of dermo-epidermal interface dermatitis, are exclusive to LE, with or without systemic disease. They are subdivided into three categories based on clinical characteristics – ACLE, SCLE and CCLE. The nonspecific lesions include other cutaneous manifestations associated with SLE. In 2004, the Düsseldorf classification added another subtype, the intermittent CLE (ICLE), which corresponds to tumid LE, previously considered as a variant of CCLE.^3^

Some limitations of the aforementioned classifications can be highlighted: a) CLE lesions cannot always be classified as acute, subacute, or chronic, based on histopathology; b) Interface dermatitis, used as a criterion to define specific CLE lesions, actually lacks specificity, as it may be present in other conditions, such as dermatomyositis, graft-versus-host disease, and drug reactions; c) Some subtypes included as specific, such as tumid LE and lupus panniculitis, do not always show interface dermatitis; d) Terms such as acute, subacute or chronic, of a chronological nature, are used to define morphological variations, in addition to being associated with ill-defined degrees of extension, such as localized or disseminated, related to topography. For these reasons, in 2010, Lipsker proposed a new classification of LE cutaneous lesions, based on clinical characteristics and histopathological findings. Specific cutaneous lesions, without the obligatory presence of interface dermatitis, are subdivided into dermo-epidermal, dermal and hypodermic. Non-specific lesions are subdivided into thrombotic, neutrophilic, or of uncertain pathogenetic nature.^11^

In the absence of a universally accepted classification, in 2013, a task force was constituted, consisting of specialists in the subject, to propose uniformity of diagnostic criteria and classification of CLE, using the Delphi method.^12^ Recently, the validation of the classification criteria for DLE, the most common form of CLE, was presented on an exclusively clinical basis. The following parameters were included, with different scores being related to skin lesions: atrophic scar (3 points), location in the pinna (2 points), preference for head and neck (2 points), dyschromia (1 point), follicular keratosis and corneal plugs (1 point), erythematous to violaceous color (1 point). A score of 5 or greater ensures 84% sensitivity and 76% specificity for classification as DLE, and the higher the score, the greater the specificity.^13^

## Etiopathogenesis

SLE and CLE are multifactorial diseases, involving a complex interaction between genetic load and environmental exposures, such as ultraviolet radiation (UVR), drugs, pesticides, and tobacco.^14^, ^15^ Epigenetic variations, such as dysregulation of gene expression, via DNA methylation, or histone modifications, caused by these external factors, may trigger the activation of innate and adaptive immunity.^4^, ^14^

Studies on genetic factors involved in CLE are still incipient compared to those described in SLE.^16^ Despite this fact, genetic polymorphisms, mutations and risk alleles have been identified in different populations of CLE, most of them associated with innate and adaptive immunity pathways.^1^, ^14^, ^17^ Genes that act in apoptosis, leukocyte migration, type I IFN pathway, complement cascade, antigen presentation, and antibody production are among the most frequently affected ones.^14^, ^17^ Genes that encode the production of pro-inflammatory cytokines are most frequently associated with innate immune pathways in CLE lesions.^1^ Examples of associations described between genes and cutaneous manifestations include *FCGR2A* (risk for ACLE), *TYK2*, *IRF5*, *TNF-α* (risk for SCLE), and *ITGAM* (risk for DLE).^15^, ^17^ HLA variants have also been correlated with skin disease progression, most notably HLA-B8, HLA-DR, and HLA-DQ.^16^ To date, only one monogenetic variant of CLE has been identified, a rare form of familial perniotic LE associated with mutations in the *TREX1* gene.^1^, ^14^, ^17^, ^18^

Among environmental factors, UVR is the most well-established trigger of CLE.^1^ Skin irradiation changes the morphology and function of keratinocytes, directly inducing the production of pro-inflammatory cytokines (IL-1α, IL-1β, IL-6, TNFα and IFNα, k and ƛ) and apoptosis.^4^, ^14^, ^17^ Increased inflammatory cytokines and exposure to cellular waste, released by cell death, trigger the recruitment of lymphocytes and plasmacytoid dendritic cells (pDC), which will trigger the immune system activation.^1^, ^18^ The pDCs are rare in normal skin and abundant in CLE lesions.^18^ Cellular waste, especially nuclear waste, is captured by pDCs, which can also constitute a reservoir of self-antigens against self-reactive B and T lymphocytes.^14^

Keratinocyte apoptosis, such as that mediated by the Fas/FasL pathway, has been shown to have a strong correlation with disease activity.^16^ The role of keratinocytes in the onset and development of CLE lesions also involves an upregulation in IFN production, especially types I and III.^1^ The produced IFN is capable of activating both the innate and adaptive immune systems, playing a central role in LE pathogenesis.^14^, ^19^

The activation of the innate immune system promotes tissue inflammation, especially mediated by pDCs and neutrophils (including neutrophil extracellular traps – NET), and increased expression of autoantigens, including Ro/SS-A (an IFN-inducible protein).^18^, ^19^ This increase stimulates the adaptive immune system, such as cytotoxic T-lymphocytes and plasma cells.^1^ Increased IFN also perpetuates the expression of cytokines and chemokines, closely related to antibody production, which preferentially deposits at the dermo-epidermal junction and result in cytotoxic insult, mediated primarily by CD8+T lymphocytes.^4^

Elevated IFN levels, especially type I, also lead directly to infiltration of Th1 lymphocytes, accelerating tissue inflammation and production of IFN-γ and IL-2, among other cytokines.^4^ The increase in these cytokines stimulates intracellular signaling pathways such as the JAK/STAT pathway and alters gene transcription.^14^

Finally, the elevation of cytokine levels, increased antigen exposure and Th1 cell activity stimulate the production of self-reactive antibodies, which are deposited mainly in the basement membrane zone and stimulate aggression by CD8+T cells and NK cells, via enzymes such as granzyme B, which induce apoptosis by activating caspases and continuing the inflammatory cycle.^4^, ^19^

The role of autoantibodies remains unclear in CLE. Patients with CCLE have a much lower occurrence of serum autoantibodies, such as antinuclear, anti-native DNA, anti-Sm, anti-Ro/SS-A, and anti-La/SS-B, when compared to patients with SLE and SCLE.^19^ On the other hand, immunoglobulins are strongly involved in the local pathogenesis of CLE lesions. IgM is the first to be recruited to the skin, subsequently attracting C3 and other immunoglobulins. Unlike SLE, where IgM alone or in combination with C3 is the most frequent form of immune complex formation, in DLE, IgG is responsible for the majority of immune complex deposits.^16^

Despite increased serum IL-17 levels and increased cutaneous IL-17A expression in SLE patients, few IL-17-producing T lymphocytes are found in DLE lesions, suggesting a less important role in this clinical manifestation.^16^ A reduction in the percentage of regulatory T lymphocytes in the skin affected by CLE has also been described. Another recent report is an inversely proportional relationship between the percentage of Th22 lymphocytes in the lesions and clinical severity scores, indicating that IL-22 can be a good indicator of skin repair.^19^

Drugs are described as another possible trigger for CLE, especially for the subacute subtype.^17^ Drugs classically recognized as CLE inducers, such as antihypertensives, antifungals, and proton-pump inhibitors, among more than a hundred drugs – have shared importance with new targeted therapies, such as immunobiologicals, immunotherapeutic and chemotherapy drugs, due to the increasing occurrence of cases related to these drugs.^4^, ^17^ It is believed that the drugs that cause CLE may directly activate the innate immune system or act indirectly, by reducing the clearance of auto-antigens.^1^ Cigarette smoking is another important risk factor associated with CLE, as it stimulates pro-inflammatory cytokines and neutrophil activation, and increases cellular stress, free radical formation, and apoptosis.^1^, ^14^, ^18^

Sex hormones seem to be a fundamental part of the pathogenesis of SLE. On the other hand, their importance in the CLE seems to be lower, especially in DLE.^16^ Despite this fact, the incidence of CLE is still higher in females. As this increase occurs even outside the fertile period, it is postulated that there may be other explanations for the higher incidence in women.^16^ The absence of significant CLE worsening during pregnancy or while using oral contraceptives also corroborates this finding.^16^ A possible alternative hypothesis to hormonal influence would be an X-linked dose effect, which could explain, for example, the increased incidence of SLE in male patients with Klinefelter syndrome (genotype XXY).^16^ Another hypothesis would be the reactivation of the inactivated X chromosome by a demethylation process in CD4+T lymphocytes.^16^

The role of the cutaneous microbiota in the pathogenesis of CLE is still poorly understood and has attracted increased attention from researchers.^16^ A significant increase in *Staphylococcus* and *Corynebacterium*, as well as a reduction in *Cutibacterium*, have been described in SLE skin lesions; however, there is yet no evidence to support a direct relationship.^16^, ^17^

## Clinical presentation and differential diagnosis

Table 2 depicts the clinical subtypes of CLE and their variants, as well as the nonspecific cutaneous manifestations that may be associated with LE.

**Table 2:** Clinical subtypes and nonspecific cutaneous manifestations of lupus erythematosus.

Subtypes of cutaneous LE	Variants
Acute cutaneous LE	Localized
	Disseminated
	TEN-like
Subacute cutaneous LE	Annular polycyclic
	Psoriasiform papulosquamous
	Rowell syndrome
	Neonatal LE
Intermittent cutaneous LE	Tumid LE
Chronic cutaneous LE	Discoid LE
	Mucosal discoid LE
	Hypertrophic/verrucous LE
	Lupus profundus/Lupus panniculitis
	Perniotic LE
	Lichen planus-like LE
	Comedonic LE
Bullous LE	‒

Type of alteration	Nonspecific manifestations
Vascular	Raynaud's phenomenon
	Livedo reticularis
	Livedo racemosa
	Vasculites
	Livedoid vasculopathy
	Degos disease type papulosis
	Cutaneous necrosis
	Splinter hemorrhage
	Thrombophlebitis
Neutrophilic	Bullous LE
	Urticarial vasculitis
	Neutrophilic urticarial dermatosis
	Amicrobial pustulosis of the folds
	Sweet syndrome
	Pyoderma gangrenosum
Undefined	Non-cicatricial diffuse alopecia
	Mucosal ulcers
	Rheumatoid nodules
	Interstitial granulomatous dermatitis
	Eruptive dermatofibroma

LE, Lupus Erythematosus; TEN, Toxic Epidermal Necrolysis.

### Acute cutaneous lupus erythematosus

It accounts for 15% of CLE cases and is always associated with SLE, usually correlating with disease activity.^3^ It is identified at the time of diagnosis in approximately 50% of SLE cases and can be triggered or exacerbated by acute sun exposure.^2^

**Figure 1:**
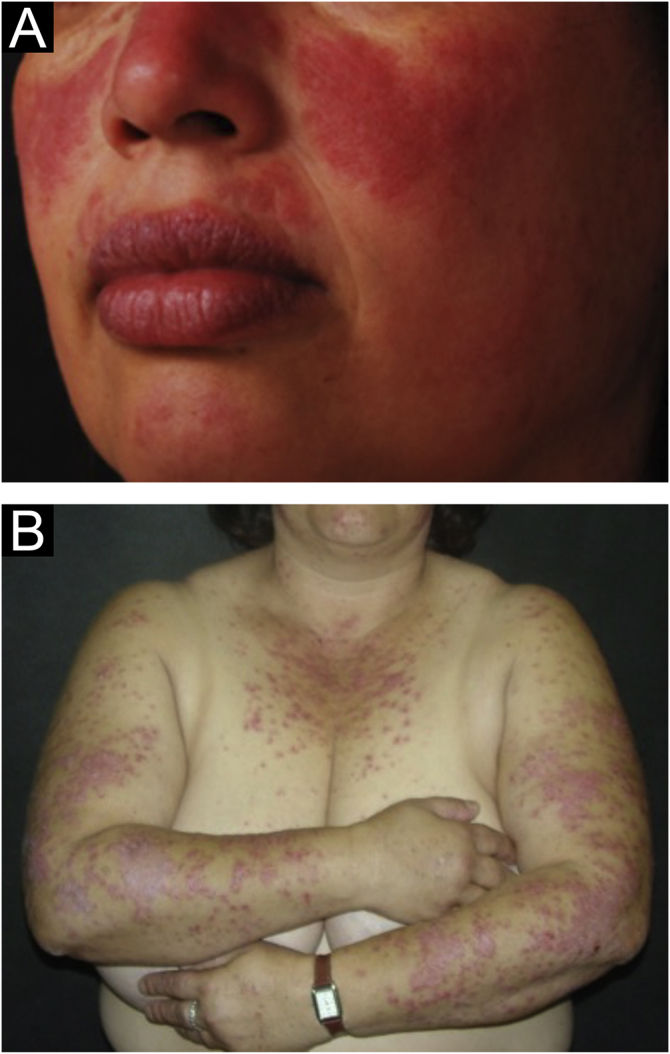
Acute cutaneous lupus erythematosus. (A) Localized form: butterfly rash on the malar regions. (B) Disseminated form: symmetrical papulosquamous eruption, affecting the trunk and upper limbs.

The localized form is the predominant one (90%‒95%) and corresponds to the typical butterfly rash, which manifests as an erythematous and finely desquamative lesion, symmetrically affecting the malar regions and the nasal dorsum, generally sparing the nasolabial folds (Fig. 1A). Facial edema may also be present. Erythema is usually transient, resolving in a few days or weeks. Eventually, it affects the forehead, chin, ears and anterior cervical region. Its main differential diagnoses are dermatomyositis and rosacea, but seborrheic dermatitis, perioral dermatitis and photoallergic contact dermatitis should also be considered. The involvement of the nasolabial folds and eyelids, as well as proximal muscle weakness, favor a diagnosis of dermatomyositis.

The disseminated form, which is less common (5%‒10%), manifests as an exanthematic urticarial or maculopapular rash, predominant in photoexposed areas (Fig. 1B). In addition to the face and neck, it symmetrically affects the V-neckline region and the extensor surface of the limbs, especially the upper limbs, but it can also extend to the trunk and even the palms and soles. When it affects the dorsum of the hands and fingers, it tends to spare the joints, contrary to dermatomyositis. It must be differentiated from drug hypersensitivity reactions, viral rashes, and phototoxic or photoallergic drug-induced dermatitis, in addition to dermatomyositis.

Mucosal involvement is frequent in both forms. The oral mucosa is the most affected one, in 8% to 45% of cases, mainly occurring in the buccal, palatine, and labial mucosa, as enanthematous or purpuric plaques, vesiculobullous lesions, erosions, and ulcers.^20^ Nasal ulcerations are less common and other mucous membranes are rarely affected.

A rare ACLE presentation, which resembles toxic epidermal necrolysis (TEN), therefore known as the TEN-like variant, is due to intense interface dermatitis with keratinocyte necrosis, which results in epithelial detachment of large areas of the skin. There may be mucosal involvement, which is often more limited and spares the ocular conjunctiva.^21^

After resolution, ACLE lesions may leave residual hyperchromia, but do not usually result in scarring, except in the TEN-like variant, or when complicated by secondary infection.

### Subacute cutaneous lupus erythematosus

**Figure 2:**
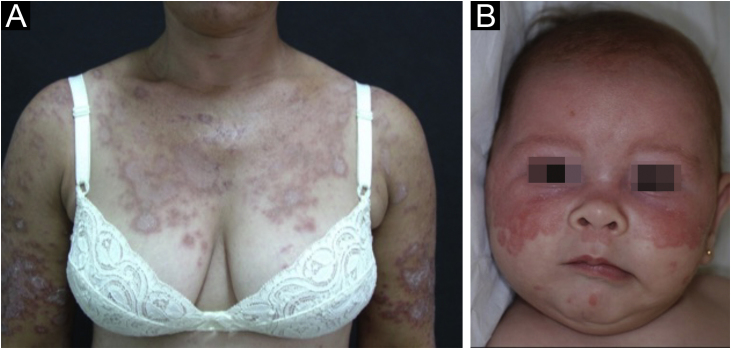
(A) Subacute cutaneous lupus erythematosus: annular, erythematous, papulosquamous plaques, confluent on the chest and upper limbs. (B) Neonatal lupus erythematosus: circular and polycyclic erythematous plaques on the face.

It accounts for about 8% of all cases of CLE, lasts longer than ACLE, and is extremely photosensitive. It has a symmetrical distribution, preferably on the exposed cervical area, upper trunk, and upper limbs, but usually spares the central region of the face.^3^ It manifests in two forms, both with erythematous plaques, one as polycyclic annular and the other as psoriasiform papulosquamous lesions (Fig. 2A), which can eventually coexist in the same patient. Atrophy and keratosis are not clinically evident in SCLE lesions. They also tend to be less edematous than ACLE lesions and less infiltrated and dyschromic than DLE ones. In both forms, vesiculobullous lesions and crusts occasionally appear at the periphery of the plaques.^6^ They usually resolve without scarring, but there may be residual hypochromia, usually temporary, but permanent in more severe forms. Eventually, ACLE or DLE lesions may coexist with SCLE lesions, but an exact differentiation between these lesions is not always possible. Oral mucosal involvement is rare, with slightly atrophic circular enanthematous plaques.^22^ It must be differentiated from granuloma annulare, erythema annulare centrifugum, erythema gyratum repens, psoriasis, and tinea corporis, among others.

About one-third of SCLE cases are triggered by drugs. In general, drug-induced SCLE is indistinguishable from idiopathic SCLE, but some particularities should lead to the suspicion of a drug-related etiology – older age, more widespread lesions, and presence of bullous or targetoid lesions.^23^ The picture is usually reversible, resolving within a few months after the withdrawal of the responsible medication. The most frequently implicated drugs are hydrochlorothiazide, calcium channel inhibitors, angiotensin-converting enzyme inhibitors, proton-pump inhibitors, terbinafine, anti-TNF agents, and anticonvulsants, but the incidence tends to vary with the development of new drugs and the changes in prescription.^23^, ^24^, ^25^ A recent review of the literature, focusing on the past decade (2010‒2020), revealed that anti-TNF agents, proton-pump inhibitors, and antineoplastic agents, particularly immune checkpoint inhibitors stand out as emerging CLE-inducing drugs.^26^ The list of responsible drugs is extensive, as shown in Table 3.

**Table 3:** Drugs reported as triggers of subacute cutaneous lupus erythematosus.

Class	Subclass	Drugs
Anti-hypertensive	Diuretics	Hydrochlorothiazide
	Angiotensin converting enzyme inhibitors	Captopril
		Cilazapril
		Enalapril
		Lisinopril
		Ramipril
	Beta-blockers	Acebutolol
		Diltiazem
	Calcium channel blockers	Nifedipine
		Nitrendipine
		Verapamil
Proton-pump inhibitors		Esomeprazole a
		Lasanoprazole
		Omeprazole
		Pantoprazole
Antifungals		Griseofulvin
		Terbinafine
Anticonvulsants		Carbamazepine
		Phenytoin
Statins		Pravastatina
		Simvastatin
Antihistamines		Brompheniramine
		Cinnarizine + tietilperazine
		Ranitidine
Antibiotics		Amoxicillin + clavulanate
		Ciprofloxacin
Nonsteroidal anti-inflammatory drugs		Naproxen
		Piroxicam
Chemotherapeutic agents		5-Fluorouracil^a^
		Capecitabine^a^
		Docetaxel
		Doxorubicin
		Gemcitabine
		Masitinib
		Mitotane
		Nivolumab
		Paclitaxel
		Palbociclib^a^
		Pembrolizumab^a^
		Tamoxifen
		Tegafur/Uracil^a^
Immunobiologicals	Anti-TNF	Adalimumab
		Etanercept
		Golimumab
		Infliximab^a^
	Anti-CD80/86	Abatacept
	Anti-CD11a	Efalizumab
	Anti-IL12/23	Ustekinumab
	Anti-IL17	Secukinumab
Antidepressants		Bupropion
Others		Alopurinol^a^
		Anastrozole
		Interferon-α and β
		Intravenous immunoglobulin
		Leflunomide^a^
		Leuprorelin
		Ticlopidine
		Tiotropium

Adapted from Borucki & Werth, 2020.^23^

a Also triggers chronic cutaneous lupus erythematosus.

The first case of SCLE associated with malignant disease was described in 1982 as lupus erythematosus gyratum repens in a patient with lung cancer. Since then, several reports have emerged, suggesting a relationship between SCLE and various solid and non-solid neoplasms, most frequently lung carcinoma and breast adenocarcinoma. In most cases, the parallelism between tumor development and dermatosis activity allowed characterizing the association as a paraneoplastic syndrome. Albeit rare, it is important to consider this possibility in SCLE with an older age onset, in patients with a higher risk for neoplasia, and in cases refractory to conventional treatment.^27^

Rowell's syndrome was originally described in 1963 as a distinct entity, due to the association of clinical findings of LE and erythema multiforme, in addition to the presence of some immunological alterations, such as speckled ANA, anti-SjT antibody and rheumatoid factor. In light of modern clinical and immunological knowledge, this syndrome should not be seen as having a nosological identity, but rather as a rare variant of SCLE, characterized by target-like skin lesions and the presence of anti-Ro/SS-A antibodies.^28^

Neonatal lupus erythematosus is also considered a variant of SCLE. It results from the transplacental passage of maternal autoantibodies against Ro/SS-A, La/SS-B and, more rarely, U1-RNP antigens. In most cases, the mother is asymptomatic but may have Sjögren's syndrome, SCLE, SLE, or other connective tissue diseases. The risk is small in the first pregnancy or in the absence of disease in previous pregnancies (2%) but increases substantially (10-fold) if a diagnosis has been made in a previous pregnancy. Skin lesions occur in up to 40% of cases of neonatal LE and may be present at birth but usually appear within the first 3 months of life, after sun exposure, and resolve spontaneously within 6 to 12 months. They are erythematous or erythematous-desquamative, circular, annular, or polycyclic and affect mainly the face, in the frontal and periorbital regions (Fig. 2B), and may extend to the scalp, but rarely affect the trunk and limbs. Seborrheic dermatitis and tinea faciei are the main differential diagnoses. Other possible manifestations, also reversible, are cytopenias (thrombocytopenia, hemolytic anemia), hepatobiliary disease, splenomegaly and, more rarely, neurological and pulmonary disorders. More severe and irreversible changes are cardiac alterations, particularly conduction disorders, including complete atrioventricular block, cardiomyopathy, and valvular heart disease, which may occur in 25% of cases and be detected before birth.^2^, ^29^

### Chronic cutaneous lupus erythematosus

Chronic cutaneous lupus erythematosus accounts for more than 70% of CLE cases and comprises several variants, some more common and others rarer.^3^

**Figure 3:**
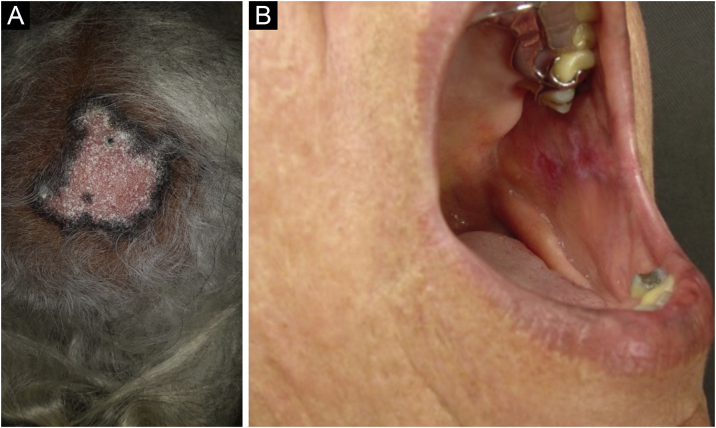
Chronic cutaneous lupus erythematosus. (A) Discoid form located on the scalp, with cicatricial alopecia. (B) Discoid form, affecting the buccal mucosa.

Discoid lupus erythematosus is the classic and most common form of CCLE, also present in 20% of SLE cases. It is predominantly located on the head and neck, but in about 30% of cases, it can be disseminated and affect the trunk and limbs, with a preference for photoexposed areas, such as the face, ear pinnae, V-neckline region, lateral cervical region, scalp and dorsal region of the upper limbs. The lesions begin as infiltrated erythematous plaques, indurated on palpation, evolving with keratosis, mainly of follicular type, atrophy and dyschromia, with hypochromia in the center and hyperchromia at the periphery. They are usually painful on palpation. They can cause vitiligoid lesions, as well as scarring, often mutilating ones, especially when they affect the nose, ear pinnae and eyelids. Lesions at different stages are often concomitant. When located in hairy areas, such as the scalp, beard, eyelashes and eyebrows, they tend to destroy the follicles, resulting in cicatricial alopecia (Fig. 3A). Palmoplantar lesions are rare, usually very painful, and can ulcerate. Exceptionally, nail and periungual lesions can be observed. In addition to solar radiation, DLE lesions can be caused by trauma, which constitutes the Koebner phenomenon.^2^, ^3^, ^30^ Drug-induced DLE is very rare, with sporadic reports associating it with leflunomide, 5-fluoruracil, capecitabine, palbociclib, pembrolizumab and most commonly with anti-TNF agents.^31^ Recent DLE lesions must be differentiated from polymorphous light eruption, sarcoidosis, pseudolymphoma, facial granuloma, and tumid LE. Chronic lesions deserve to be differentiated from lupus vulgaris, hypertrophic actinic keratosis, squamous cell carcinoma and keratoacanthoma.

Mucosal discoid lupus erythematosus is reported in 3% to 25% of patients with DLE and is usually asymptomatic in 25% of the cases. It classically presents as enanthematous plaques, with keratotic papules, central atrophy or erosion and radiated or reticulated keratosis at the periphery. It may also manifest as striated or homogeneous white plaques, erosions and ulcerations. It is more common in the oral mucosa, mainly in the buccal mucosa (Fig. 3B), palatine region and labial mucosa, but it can also affect the gums and tongue. The nasal, genital and anal mucous membranes can also be affected by discoid lesions. The lesion in the ocular conjunctiva is characteristic and more common in the lower palpebral margin, which can result in scarring, loss of eyelashes, and ectropion. There is a risk of malignant transformation into squamous cell carcinoma, most frequently on the lips.^2^, ^22^, ^32^ Its main differential diagnosis is oral lichen planus.

**Figure 4:**
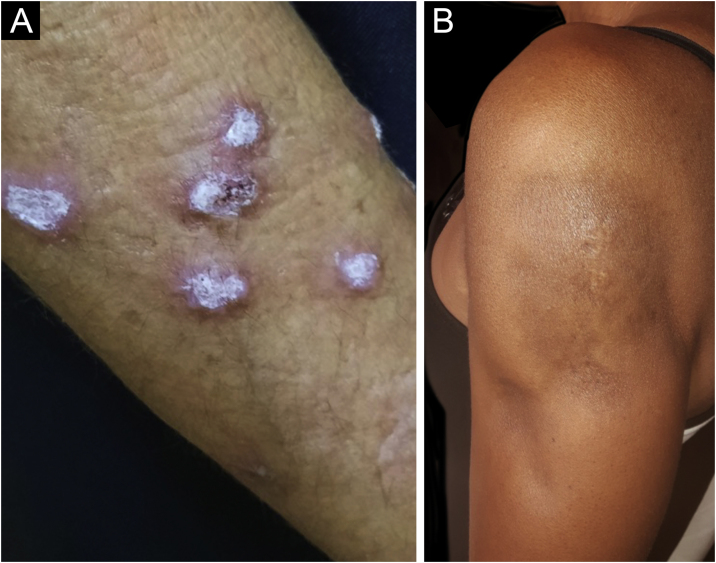
Chronic cutaneous lupus erythematosus. (A) Verrucous variant, located on the upper limb. (B) Lupus panniculitis, affecting the arm and progressing to lipoatrophy.

Hypertrophic lupus erythematosus, also known as verrucous LE, is a very rare variant of CCLE that manifests as erythematous, papular, or nodular lesions, with a keratotic and somewhat verrucous surface, located mainly in the extensor regions of the upper limbs (Fig. 4A) and eventually on the face and upper trunk. Typical discoid lesions often coexist, which facilitates the diagnosis.^2^ It must be differentiated from hypertrophic lichen planus, keratoacanthoma and common warts.

Lichen planus-like lupus erythematosus or lupus erythematosus-lichen planus overlap syndrome refers to the combination of clinical, histopathological and immunopathological findings of both dermatoses. It is a very rare condition, with a chronic evolution and predominant in females, presenting as painful, desquamative, bluish-red plaques with an atrophic center, located on the upper extremities and less frequently on the legs, face and trunk, with palmoplantar involvement being characteristic. Hypertrophic lesions can also occur. There are also reports of mucosal involvement, nail dystrophy and cicatricial alopecia, as well as cases triggered by drugs.^2^, ^33^, ^34^

Lupus profundus or lupus panniculitis accounts for only 2% to 3% of CCLE cases, is more frequent in adults and females, and manifests as indurated subcutaneous plaques and nodules, adhered to the overlying skin, usually painful, located mainly on the face, shoulders, arms, thighs, gluteal region and breasts (Fig. 4B). The surface skin may or may not have DLE lesions. It usually causes intense atrophy of the subcutaneous tissue, leaving depressed areas with aesthetic disfigurement, especially when located on the face. Eventually, there are calcifications and ulcerations. The disease has a chronic course, intermingled with periods of exacerbation and remission.^2^, ^30^ It must be differentiated primarily from other types of panniculitis, panniculitis-like subcutaneous T-cell lymphoma, and subcutaneous sarcoidosis.

Perniotic lupus erythematosus is a rare form of CCLE, which resembles perniosis and manifests as painful, papular, nodular or edematous lesions, with an erythematous violaceous color. They may develop into erosion or ulceration. They are triggered by exposure to cold and usually affect the fingers and dorsum of the hands. Toes, plantar regions, and heels can also be affected. The nose and ear pinnae are less frequently affected. Raynaud's phenomenon may be present in some cases. The condition can coexist with DLE lesions or occur in the context of SLE. It is estimated that 20% of patients with perniotic LE will develop systemic disease. Some immunological alterations can be observed, such as hypergammaglobulinemia, positive rheumatoid factor and ANA, as well as specific antiphospholipid and antinuclear antibodies, mainly anti-Ro/SS-A.^35^ There is a familial form of the disease, which manifests in childhood, of autosomal dominant inheritance, related to mutations in the *TREX1* gene and less frequently in the *SAMHD1* or *TMEM173* genes.^2^, ^36^ The main differential diagnosis is idiopathic perniosis, but lupus pernio, an acral form of sarcoidosis, should also be considered as well as vasculitides and acral vasculopathies.

Comedonic lupus erythematosus is another rare variant of CCLE, which manifests as comedones, papules, infiltrated erythematous plaques, and cysts in seborrheic and photoexposed areas, leaving depressed acneiform scars. It preferentially affects young and middle-aged women, with smoking being an important risk factor, as in other forms of CCLE. It can be accompanied by typical DLE lesions, as well as associated with SLE. It is often confused with acne vulgaris, nevus comedonicus, and nodular elastoidosis with cysts and comedones, which often delays diagnosis and treatment, resulting in inaesthetic scars.^37^, ^38^, ^39^

### Intermittent cutaneous lupus erythematosus

**Figure 5:**
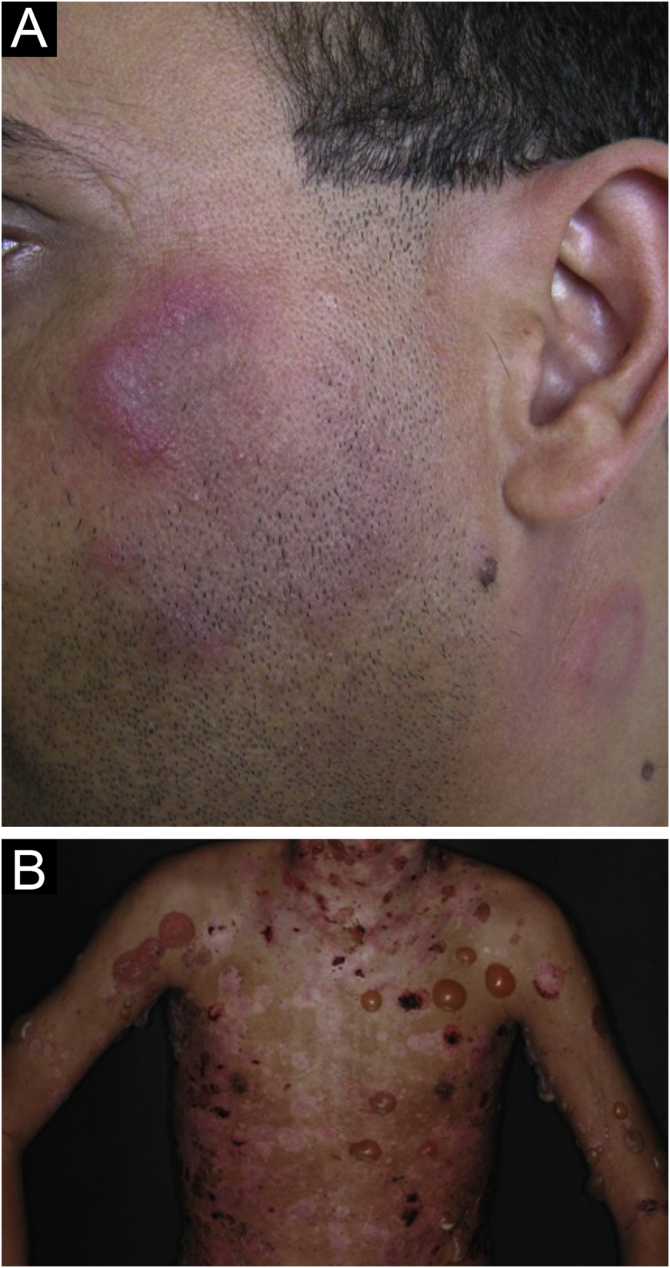
(A) Lupus erythematosus tumidus: arciform and annular erythemato edematous lesions on the face and neck. (B) Bullous lupus erythematosus: bullous, hemorrhagic ulcerocrusted and hypochromic residual lesions located in the cervical region, trunk, axillae and upper limbs.

Previously categorized among the CCLE variants, tumid lupus erythematosus was reclassified as intermittent cutaneous lupus erythematosus due to its clinical course, in which periods of remission and recurrence alternate. It is a rare variant of CLE, which affects more women (60% of cases), but the preponderance over men is less evident than in other forms of CLE. A striking characteristic of tumid LE is exacerbated photosensitivity, perhaps the most intense among all forms of LE. It manifests as single or multiple, erythemato edematous, smooth-surface plaques that can show an annular or arcuate configuration (Fig. 5A). They are located in photoexposed areas, mainly on the face, cervical area, upper trunk and upper limbs. It is usual for the lesions to resolve spontaneously within a few weeks, causing no dyschromia or scarring but episodes with new lesions occur. The occurrence of the isolated form is more common, but it may be associated with other forms of CLE, mainly DLE, and rarely with SLE. Immunological alterations are also unusual, such as ANA and specific antinuclear antibodies. It must be differentiated from polymorphous light eruption, early DLE, reticular erythematous mucinosis, and cutaneous pseudolymphomas such as Jessner lymphocytic infiltration, considered by many authors as being tumid LE itself.^2^, ^40^, ^41^

### Bullous lupus erythematosus

Bullous LE is an autoimmune subepidermal bullous dermatosis, always associated with SLE, mediated by autoantibodies against collagen VII. It manifests as tense vesicles and bullae (Fig. 5B), which appear suddenly on healthy skin or on erythematous and infiltrated plaques, eventually showing an annular configuration. They show a predilection for the face, upper trunk, neck, supraclavicular regions, and axillary flexures, but can spread to other nonexposed areas. They are not usually accompanied by CLE-specific lesions, except for malar erythema, which may appear later, during disease evolution. The oral and genital mucosa may be affected, with typical labial and perioral lesions.^42^ It is more common in black women, between the second and fourth decades of life. Bullous LE develops before, concomitantly, and, less frequently, after the diagnosis of SLE, and may be a marker of intense systemic activity, with an increased risk of lupus nephritis and neuropsychiatric manifestations.^43^ The lesions develop into erosions and crusts, regressing without scars and milia, but may cause residual dyschromia. Recurrence is uncommon, even with the persistence of active systemic disease.^44^ It must be differentiated from epidermolysis bullosa acquisita, linear IgA bullous dermatosis, dermatitis herpetiformis, and bullous pemphigoid.

### Nonspecific cutaneous manifestations of lupus erythematosus

Excluding the previously presented clinical subtypes and respective variants, all other cutaneous manifestations associated with LE must be considered nonspecific. They are associated with SLE and are generally related to disease activity. However, they are not exclusive to SLE and may occur in other diseases, usually autoimmune or autoinflammatory ones.^45^, ^46^, ^47^ Bullous LE, although classified as a nonspecific, neutrophilic manifestation associated with LE, should be considered a specific clinical subtype of CLE, as it is always associated with SLE.^11^ The most common nonspecific manifestations are listed in Table 2.

## Diagnosis

The diagnosis of CLE is based on data collected in the anamnesis and physical examination, together with histopathological findings and, eventually, immunohistopathology of the cutaneous lesions, aiming to define the clinical subtype.

The type and scope of the laboratory investigation must be tailored to each individual patient, depending on the subtype of CLE which is defined based on clinical and histopathological findings.

Routine biochemical tests should be performed to help identify a possible systemic disease, in addition to specific tests, according to the proposed treatment, before starting the medication and to monitor its potential adverse effects. Additional tests may be necessary after confirming the diagnosis and defining the CLE subtype, including serological tests to characterize the autoantibody profile and tests to evaluate the systemic activity of the disease, as well as complementary tests to investigate the involvement of specific organs, which may aid in determining the prognosis. The main laboratory tests recommended in CLE are listed in Table 4. In cases of SLE, the intervention of a rheumatologist and, eventually, other specialists may be necessary to guide the individualized complementary propaedeutics for each patient.^48^

**Table 4:** Recommended laboratory evaluation in cutaneous lupus erythematosus.

**Routine exams** (suspected CLE)
Whole blood count
Erythrocyte sedimentation rate
C-reactive protein
ANA (HEp-2)
Liver enzymes (AST, ALT, AP, GGT)
Kidney function (urea, creatinine)
Urinalysis
**Special exams** (confirmed CLE)
Specific antibodies (anti-native DNA, -Sm, -Ro/SS-A, -La/SS-B, -RNPn)
Serum complement (C3, C4)
Antiphospholipid antibodies (IgG and IgM anticardiolipin antibodies; Lupus anticoagulant; β2-glycoprotein 1)
Lupus anticoagulant; β2-glycoprotein I)
Rheumatoid factor
Immunoglobulins (immunoelectrophoresis)
TSH, T4, antithyroid antibodies
24 h urinary protein
Creatinine clearance
Glucose-6-phosphate dehydrogenase

Adapted from Kuhn et al., 2014.^48^

It is important that the definition of systemic disease is not strictly dependent on the diagnostic criteria included in the classification systems, such as the SLICC 2012 and the EULAR/ACR 2019, which were primarily developed with the objective of obtaining diagnostic uniformity in the selection of patients for clinical trials, since they are not able to cover all the manifestations that can occur in SLE.^30^

The use of disease activity scores, originally created to measure outcomes in clinical trials, is recommended in clinical practice, as they allow a more objective analysis of disease progression during follow-up of patients with CLE, and can be used as a parameter when assessing response to the prescribed treatment.

### Histopathology

Different forms of CLE, with the exception of lupus profundus and tumid LE, share histopathological findings, making a thorough clinic opathological correlation indispensable for defining the subtype. Histopathological differentiation depends on the evolution and stage of the lesions. Thus, from the histopathological point of view, LE could be classified as recent (ACLE, SCLE and early DLE), fully developed (DLE), and late (atrophic-scarring DLE). The alterations tend to be more subtle in recent lesions, being quite evident in fully developed lesions.^19^, ^49^

The main findings are the perivascular and periadnexal lymphocytic inflammatory infiltrate in the superficial and deep dermis, as well as interface dermatitis, characterized by the aggression of lymphocytes to the dermo-epidermal junction. As a consequence, other changes occur, such as vacuolar degeneration of the basal layer and keratinocyte necrosis in the lower layers of the epidermis, followed by basement membrane thickening. The epidermis becomes thinned and the epithelial ridges become flattened. Mucin deposition in the dermis is a typical finding of LE, although non-specific, varying in intensity according to the type of lesion.^47^

In DLE, the prototype of fully developed lesions, in addition to the alterations already described, the findings of hyperkeratosis, follicular corneal plugs and epidermal thinning are very evident. The late, cicatricial stage of DLE shows pigmentary incontinence, vascular ectasia, dermal fibrosis, and adnexal loss.^19^, ^47^

In ACLE, the alterations are usually milder, and there may be edema and hemorrhage in the superficial dermis. The lymphocytic infiltrate is slight, perivascular and superficial only, with the presence of neutrophils in the most recent lesions. The TEN-like variant of ACLE shows severe hydropic basal degeneration, which results in dyskeratosis, subepidermal cleavage, and complete necrosis of the epidermis.^19^, ^47^

In SCLE, the interface dermatitis is usually intense, with many cytoid bodies. The lymphocytic infiltrate is superficial and predominantly perivascular. Epidermal thinning, hyperkeratosis, follicular plugs, mucin deposition, and basement membrane thickening are less prominent than in DLE.^19^, ^47^

Hypertrophic LE, in addition to the alterations observed in DLE, shows acanthosis and marked pseudoepitheliomatous hyperplasia, besides intense hyperkeratosis.^47^, ^49^

Lupus profundus presents as a lobular lymphocytic panniculitis, with paraseptal lymphoid nodules and hyaline necrosis of adipocytes, presence of plasma cells, in addition to mucin deposition in the reticular dermis and occasionally in the hypodermis. Fibrosis and calcification can be seen in the final stage lesions. The epidermal and dermal alterations, which are characteristic of LE, may be present in half of the cases.^47^, ^49^

Tumid LE is characterized by intense perivascular lymphocytic infiltrate in the superficial and deep dermis, in addition to abundant mucin deposition, without the presence of epidermal alterations and interface dermatitis.^47^, ^49^

In perniotic LE lesions, in addition to the characteristic LE findings, the presence of lymphocytic vasculitis, eventually, with fibrinoid necrosis and thrombosis, edema of the papillary dermis and perieccrine lymphocytic infiltrate stands out, to a lesser extent than in DLE.^47^, ^49^

Bullous LE shows a predominant neutrophilic infiltrate, which is often aligned with the dermoepidermal junction and generates microabscesses in the dermal papillae, in addition to subepidermal cleavage and bullae with neutrophils inside them.^47^, ^49^

### Immunohistopathology

Direct immunofluorescence (DIF), performed on sections of tissue obtained by biopsy of the skin lesion, aims to identify immune deposits at the dermo-epidermal junction and is also known as the lupus band test (LBT). IgG, IgM, IgA and C3 immune deposits are usually the ones searched for. The LBT is considered positive when there is deposition of granular material in a band along the basement membrane zone. It is a test that can help the diagnosis, if the histopathology is not conclusive in the presence of lesions suggestive of LE, although it is not specific. It can also be observed in other dermatological conditions, such as dermatomyositis, and even in normal or photodamaged skin, mainly on the face.^48^, ^50^ The type of deposit varies according to the chronology and topography of the lesion, as well as the biopsy site, whether in the center or periphery of the lesion. IgM and IgG are the most commonly detected immune deposits, in association or not with C3. IgA deposition is less common. LBT is typically positive in lesional skin of nearly 100% of ACLE cases, in about 60% of SCLE cases, and in 90% of DLE cases.^11^, ^19^, ^47^

A recent study that evaluated 2,050 skin biopsies submitted to histopathological analysis and direct immunofluorescence, with diagnostic hypotheses of different dermatoses, concluded that the value of DIF in aiding the diagnosis of LE is questionable and its use is not recommended as routine.^50^ Although a positive LBT test in non-lesional, unexposed skin is highly specific for SLE, it adds little information to findings obtained jointly with the clinical examination, histopathology, and serology. Moreover, its association with the presence of anti-native DNA antibodies in serum has been demonstrated, albeit with similar sensitivity and specificity rates, suggesting that the use of both tests is redundant.^50^

In bullous LE, DIF of the perilesional skin demonstrates continuous deposition, with a linear or granular pattern, along the basement membrane zone, mainly of IgG, in addition to IgM, IgA, and C3. Indirect immunofluorescence using the salt-split skin technique reveals deposits on the dermal side of the cleavage.^42^, ^47^

### Antinuclear antibodies

Antinuclear autoantibodies are immunological markers used both in disease diagnosis and monitoring of LE. The most used screening test is the antinuclear antibody (ANA), an indirect immunofluorescence technique, having HEp-2 cells as substrate. ANA is more relevant in ACLE/SLE, being demonstrated in almost all patients (94% to 100%), usually at high titers, greater than 1/160. However, even at high titers, they are not specific for SLE, as they can be detected in many diseases, such as other connective tissue diseases, hematological and liver diseases, viral infections, during use of several medications, and even in healthy individuals.^51^ ANA is demonstrated to a lesser extent in other forms of CLE, in 52% to 80% of SCLE patients and 5% to 17% of DLE patients.^52^

As it is not a specific test for LE, it is crucial that, in the presence of positive ANA, the specificity of the antinuclear antibody be determined through additional tests.

Anti-native DNA and anti-Sm antibodies are the most relevant ones, because they are specific for SLE, although they have a lower sensitivity of 56% to 70% and 19% to 25%, respectively. In addition to diagnostic value, the anti-native DNA antibody can be used to monitor the disease, since its serum levels tend to reflect disease activity, especially nephropathy, mainly when there is concomitance with anti-Sm antibody.^19^, ^51^ The prevalence of anti-native DNA and anti-Sm antibodies are low in SCLE patients and virtually null in CCLE patients.^19^, ^25^

Anti-Ro/SS-A and anti-La/SS-B antibodies are not specific for LE and are often associated with Sjögren's syndrome. They occur, respectively, in 36% to 64% and 8% to 33% of SLE patients and are related to cutaneous and hematological manifestations, such as cytopenias. Anti-Ro/SS-A and anti-La/SS-B antibodies are present in between 70% to 90% and 30% to 40%, respectively, of SCLE cases, and in up to 25% and 5%, respectively, of DLE cases. Particularly, anti-Ro/SS-A antibodies are considered markers of SCLE and are related to the extreme photosensitivity of this subtype of CLE. Moreover, when present in the pregnant woman, these antibodies cross the placental barrier and can cause neonatal LE. Antibodies specifically directed against the 52 kD subunit of the Ro/SS-A antigen are associated with an increased risk of congenital heart block. Adults with these antibodies may also have QT prolongation, with an increased risk of developing ventricular arrhythmias.^51^

Anti-RNP antibodies are characteristic of mixed connective tissue disease but may be present in 23% to 49% of SLE patients and do not correlate with any disease manifestations.^51^ They may occur in 8% to 10% of SCLE cases and very rarely in CCLE.^52^

### Assessment of disease activity and skin damage

Patients diagnosed with CLE should undergo a complete dermatological examination, not only during episodes of disease exacerbation but also at regular intervals, aiming at assessing disease activity and progression, as well as identifying possible damage resulting from cutaneous involvement.

Several disease activity scores are well established in the evaluation of patients with SLE, for use in clinical trials and in daily practice, such as the SLEDAI (Systemic Lupus Erythematosus Disease Activity Index). Despite including some dermatological criteria, these scores lack the sensitivity to assess cutaneous activity in different CLE subtypes.^12^

For this reason, the CLASI (Cutaneous Lupus Area and Severity Index) was created, an instrument specifically developed to quantify cutaneous involvement in LE, with Activity (CLASI-A) and Damage (CLASI-D) scores. This instrument evaluates the morphology (erythema, scaling/keratosis, dyschromia and scarring/atrophy) and the anatomical location (13 sites) of the skin lesions, as well as the involvement of mucous membranes and scalp. It was originally established as a resource to measure outcomes in clinical trials and validated by dermatologists and rheumatologists. CLASI also showed good correlation with indicators of patient quality of life and showed to be a useful and easy-to-apply instrument in clinical practice.^11^, ^12^, ^53^

Subsequently, the revised CLASI (RCLASI) was developed, which added new parameters – edema/infiltration and subcutaneous plaques/nodules – to improve the accuracy in assessing the activity of the cutaneous disease, covering important aspects of some subtypes of CLE, such as tumid LE and lupus profundus.^48^

However, a recent systematic review found that, among the trials that used the CLASI as a measure of the outcome of therapeutic interventions, there are few with high-quality evidence. This study concluded that additional validation is needed to assess the effectiveness of CLASI in the assessment of different subtypes of CLE. The authors suggest that the use of standardized outcome measures, reported by the patient and the physician, could reduce the heterogeneity and allow comparisons between patients included in different clinical trials. They also recommend that the CLASI-50 (50% reduction in the CLASI-A score) could be more effective as a measure of therapeutic response than the CLASI-20 (20% reduction in the CLASI-A), generally used as an indicator of good response in clinical trials.^54^

## Progression from cutaneous lupus to systemic lupus erythematosus

A total of 5% to 25% of cases of isolated CLE, regardless of the subtype, may progress to SLE during its evolution, with a mean time of eight years between the diagnosis of the cutaneous disease and the development of the systemic one. A population-based study conducted in Sweden showed that the greatest possibility of developing SLE occurs up to 3 years after the diagnosis of the skin disease, in addition to being more likely in the female sex.^52^

Conversely, approximately 50% to 60% of SCLE patients experience transition or can already be classified as SLE at the time of diagnosis of the cutaneous disease, usually meeting cutaneous, musculoskeletal, and serological criteria.^52^

However, CLE patients which progress to SLE tend to have milder systemic symptoms, with rare cardiopulmonary and neurological manifestations, as well as other severe complications of the disease. The criteria related to nephropathy are relevant indicators for differentiating between isolated cutaneous disease and systemic disease. When present, kidney involvement tends to be less severe in those who progress from CLE to SLE, but more consistent studies are still necessary to support this conclusion.^52^

There is evidence that DLE and SCLE with disseminated lesions are more likely to develop systemic manifestations than those with localized lesions. A retrospective study, which compared DLE patients with localized disease and DLE and SCLE patients with disseminated disease, showed that 30% of cases with disseminated lesions had extracutaneous manifestations, such as nephritis, pleuritis, and polyarthritis, while those with the localized disease had no systemic manifestations.^52^

Nonspecific skin lesions are prevalent in SLE, especially with active disease. Therefore, when present, they imply a greater probability of systemic disease, particularly the occurrence of periungual telangiectasias. The presence of these lesions was seen in 76% of SLE patients with concomitant DLE and in no patients with exclusive DLE.^52^

Patients with CLE with the potential to develop SLE are more likely to have ANA at high titers, compared to those with exclusive CLE. In addition to being markers of relevant systemic involvement in SLE patients, high ANA titers and the presence of specific anti-native DNA or anti-Sm antibodies are also indicative of progression to systemic disease in patients with CLE.

Other persistent laboratory abnormalities, such as anemia, leukopenia, thrombocytopenia, and increased erythrocyte sedimentation rate, are also considered important markers of progression to SLE.^52^

It is also a matter of debate whether the early administration of antimalarials in patients with CLE, with the potential to develop the systemic disease, could prevent the progression to SLE or attenuate damage to vital organs, once the systemic disease is installed.^52^

Periodic follow-up is recommended, at shorter intervals, for patients with CLE who have risk factors for progression to SLE, with a thorough clinical examination and adequate laboratory review.^52^

## Treatment

Treatment of CLE involves pharmacological and non-pharmacological measures. Choosing the most effective therapy for each case can be challenging and requires attention to clinical manifestations and familiarity with available therapies. It is important to assess patient adherence to treatment at each medical visit.^55^ Although there are only three drugs approved by the US Food and Drug Administration (FDA) for use in SLE – corticosteroids, hydroxychloroquine, and belimumab – and none specifically approved for CLE, it is possible to find data in the literature that allow rationalizing the therapeutic approach.^56^

### General measures

Photoprotection is a fundamental pillar of the treatment of CLE, as sunscreens can prevent the appearance of lesions in patients with CLE.^1^, ^55^, ^56^, ^57^, ^58^ However, other patient care measures are also needed, such as behavioral changes, and wearing hats and long-sleeved garments, preferably with UVR skin-protection technology.^6^, ^58^, ^59^

Smoking cessation should be ascertained and encouraged at each visit, preferably with a referral of the patient to support programs and therapies.^6^, ^57^ Vitamin D supplementation in patients with deficiency may be beneficial for disease control.^56^, ^57^, ^60^ In the case of drug-induced CLE, the suspected medication should be promptly discontinued.^6^

Contraception may be necessary when using teratogenic drugs. Women with CLE can use combined oral contraceptives if there is no history of thromboembolism or high levels of antiphospholipid antibodies; otherwise, an intrauterine device or the use of isolated progestogens should be chosen.^45^, ^61^

The use of cosmetic camouflage and hair protheses can hide scars and alopecia, improving patients quality of life and self-esteem.^59^, ^62^ It is important that patients with CLE be advised to avoid interventions that traumatize the skin, due to the risk of koebnerization.^62^

### Topical treatment

Corticosteroids are considered the first line of topical treatment due to their anti-inflammatory effect.^1^, ^6^ They can be used in cases of localized lesions or as adjuvant therapy in patients on systemic treatment. Potent corticosteroids such as clobetasol, are more effective in controlling the disease than low-potency ones.^56^, ^61^ However, these medications are associated with a higher risk of side effects, such as striae and telangiectasias due to their effect on fibroblasts and blood vessels, respectively, in addition to rosaceiform perioral dermatitis.^6^, ^60^ Therefore, potent topical corticosteroid therapy should be used for the shortest possible time. Intralesional corticosteroid injections may be used for localized hypertrophic lesions.^6^, ^55^

Topical calcineurin inhibitors – tacrolimus 0.03% or 0.1% ointment and pimecrolimus 1% cream – can be used as a substitute for corticosteroids in cases requiring prolonged treatment or with a higher risk of side effects, such as lesions on a child’s face.^55^, ^61^ They are less effective than potent topical corticosteroids.^1^ Side effects related to the use of these medications include burning sensation, pruritus, and erythema at the site of the application.^57^ Some studies have shown good results with the combined use of clobetasol 0.05% and tacrolimus 0.03%.

R-salbutamol is a β2-adrenergic receptor agonist that inhibits IL-2 and IFN-γ production and may improve CLE lesions when used in a 0.5% cream.^55^, ^60^ However, it is not commercially available for topical use.^60^ Topical retinoids have been successfully used in some small case series.^60^

### Systemic treatment

Patients with localized lesions refractory to topical treatment or with disseminated lesions usually require systemic treatment.^61^ Table 5 shows the level of evidence and the degree of recommendation of the main drugs for systemic use in the treatment of CLE.

**Table 5:** Level of evidence and degree of recommendation of the main drugs for systemic use in cutaneous lupus erythematosus.

Drug	Level of evidence^a^	Degree of recommendation^b^
Hydroxychloroquine	1	A
Acitretin	2	B
Isotretinoin	2	B
Methotrexate	4	
Dapsone	4	
Thalidomide	2	A
Mycophenolate mofetil	2	B
Azathioprine	4	
Belimumab	2	B

Adapted from Yan et al., 2020.^58^

a Level of Evidence: 1 – Randomized Clinical Trial (RCT), Systematic Reviews (SR)/RCT meta-analysis; 2 – SR of cohort studies, cohorts; 3 – SR of case-control studies, case-control studies; 4 – case series and low-quality cohort and case-control studies; 5 – expert opinion.

b Degree of recommendation: A ‒ more consistent observational or experimental studies (meta-analyses or RCT); B – less consistent observational studies (other non-randomized or observational clinical trials and case-control studies); C ‒ case reports or series (uncontrolled studies); D – opinion devoid of critical evaluation, based on consensus, physiological studies or animal models.

### Antimalarials

Antimalarials (AM) are the first line of systemic treatment and are likely to prevent the progression of CLE to systemic disease.^6^, ^59^, ^61^, ^63^ They are capable of inhibiting antigen presentation by pDC, the formation of antigen-antibody complexes, and signaling via toll-like receptors, reducing the production of type I IFN.^14^, ^55^, ^59^ Hydroxychloroquine (HCQ) is the most used AM due to its better safety profile regarding ocular toxicity when compared to chloroquine (CQ). The recommended dose of HCQ, in part of the literature, is 6.5 mg/kg/day.^55^ However, in 2016, the American Academy of Ophthalmology recommended that doses greater than 5 mg/kg/day of HCQ and 2.3 mg/kg/day of CQ be avoided, due to the increased risk of retinopathy.^58^

The AM response rate is about 63% among the various CLE subtypes and may be slightly higher for CQ when compared to HCQ.^64^ The average response rate was 91% in ACLE cases, 57% in DLE, and only 31% in perniotic LE.^64^ CCLE patients tend to respond more slowly to AM than ACLE patients.^56^

In refractory cases, one AM can be substituted for another – HCQ for CQ or CQ for HCQ; in these cases, the response rate to the second AM can reach 56% but drops to 42% after one year and 22% after two years.^4^, ^6^, ^45^, ^60^^,^^64^ The addition of quinacrine, a drug not available in Brazil, at a dose of 100 to 200 mg/day, in cases refractory to AM as monotherapy, can increase the response rate to 66%, without increasing the risk of retinopathy.^45^, ^57^, ^58^, ^61^^,^^64^ The use of AM during pregnancy and lactation is recommended, especially in patients with skin lesions and SLE, and may reduce the risk of cardiac involvement from neonatal LE.^65^

The side effects of AM include nausea, vomiting, skin pigmentation, dizziness, headache, ototoxicity, and peripheral neuropathy.^6^ Retinopathy is the most relevant side effect and occurs in up to 1% of cases.^6^ Patients should be evaluated at baseline and, if they have no additional risk factors, annually after the fifth year of drug use.^61^ Patients taking HCQ at doses above 5 mg/kg/day, with renal impairment, on concomitant use of tamoxifen, or with pre-existing retinal maculopathy are at increased risk for retinopathy and should be monitored more frequently.^56^ Campimetry and fundus examination might not detect the early alterations, and optical coherence tomography is recommended to aid in early diagnosis.^65^ There have been rare reports of cardiac toxicity, with QT prolongation.^65^

When available, serum HCQ measurements can be used to assess treatment after six months of nonresponsive use.^45^, ^60^ Values below 200 ng/mL indicate poor adherence to treatment, while values above 750 ng/mL are correlated with better rates of disease activity control.^45^, ^56^ Patients with contraindication to AM or with an optimized dose of AM but no response or with partial response have an indication for second-line treatment drugs. Smokers are more likely to be nonresponsive to AM.^57^

### Methotrexate

Methotrexate (MTX) is the first choice among second-line drugs for patients who are refractory or have any contraindication for AM use.^55^, ^66^ It is a dihydrofolate reductase inhibitor, which affects cell replication and suppresses antibody production.^59^ The recommended dose can range from 7.5 to 25 mg a week, orally or subcutaneously. The side effects include nausea, vomiting, abdominal pain, hepatotoxicity, mucosal ulceration, and bone marrow suppression.^4^, ^55^, ^60^ Subcutaneous application and the administration of folic acid on the days following medication use can significantly reduce gastrointestinal side effects.^58^, ^61^ The patients should be laboratory-monitored in the first few weeks of drug use, after increasing doses, and quarterly during regular follow-up.

The use of MTX should be avoided by alcoholic patients, those in concomitant use of hepatotoxic drugs, with severe hepatic steatosis, renal failure, or underlying liver disease, including viral hepatitis, conditions that should be investigated before starting the drug.^58^, ^61^ The risk of hepatotoxicity outside these conditions is low.^61^ Interstitial pneumonitis is a rare and potentially fatal complication.^4^, ^55^ MTX is teratogenic and adequate contraception should be recommended.^60^

### Systemic retinoids

Retinoids are used successfully in the treatment of refractory CLE, especially the verrucous forms.^6^, ^55^, ^61^ They inhibit the production of pro-inflammatory cytokines such as IL-6 and IFN-γ and regulate and normalize keratinocyte differentiation.^55^ Literature data show no significant difference in efficacy between HCQ and acitretin in patients with different CLE subtypes.^57^ Isotretinoin has also been used in small case series.^6^

The acitretin and isotretinoin dose is 0.2 to 1 mg/kg/day. Response is usually rapid and occurs within two to six weeks. Recurrence also often follows shortly after medication withdrawal.^61^

Patients using retinoids should be monitored regularly for the risk of hepatotoxicity and increased serum triglyceride levels.^6^ Other side effects include mucocutaneous xerosis and bone changes such as hyperostosis.^67^ The use of sunscreens should be intensified, due to the risk of worsening photosensitivity.^67^ Due to the risk of teratogenicity, women of childbearing age should be placed on adequate contraception during and after treatment completion (isotretinoin, up to one month, and acitretin, up to two to three years).^6^, ^55^

### Dapsone

Dapsone is an immunomodulatory and antimicrobial agent that inhibits myeloperoxidase present in neutrophils and monocytes.^6^, ^59^, ^68^ It can be used alone or in combination with AM. More than 50% of patients with CLE respond favorably to the use of dapsone, including those with DLE, a classically more resistant subtype, where the response rate approaches 60%.^6^, ^68^ Dapsone is considered the drug of first choice in the treatment of bullous LE and other neutrophilic manifestations of LE, such as urticarial vasculitis.^56^, ^61^ Hyperkeratotic variants usually do not respond well to dapsone.^55^

The initial dose is 50 mg/day and can be increased up to 200 mg/day.^55^, ^61^ Patients should be evaluated for glucose-6-phosphate dehydrogenase deficiency before starting treatment.^58^

Side effects can be severe, such as drug rash with eosinophilia and systemic symptoms (DRESS syndrome), methemoglobinemia, and agranulocytosis.^55^, ^57^ Hemolytic anemia may occur in up to 50% of patients.^57^, ^68^ Hemoglobin levels should be monitored during the first month of treatment and every three months, subsequently. Methemoglobin levels can be assessed between the 8^th^ and the 14^th^ day after introducing the medication.^60^ Dapsone is the only second-line drug that can be used during pregnancy and breastfeeding.^60^, ^61^

### Mycophenolate mofetil

Mycophenolate mofetil (MMF) is considered a third-line agent for the treatment of CLE.^55^ MMF causes guanosine triphosphate depletion, required for lymphocyte and monocyte adhesion to the endothelium during the inflammation process, in addition to inducing T-lymphocyte apoptosis and reducing B-lymphocyte activation.^55^ A complete or significant response rate has been reported in 62% of CLE patients.^6^ It can be used alone or in combination with AM,^4^ and the starting dose is 500 mg/day, which can be increased up to 3 g/day.^61^

The most frequent side effects are gastrointestinal ones, cytopenias, hepatotoxicity, and viral and urinary infections.^55^ Patients should have monthly laboratory assessments.^61^ The drug is category X and cannot be used during pregnancy.^55^

### Azathioprine

Azathioprine is a purine analogue that depresses T- and B-lymphocyte function and reduces antigen presentation.^59^ It may be indicated in CLE in case of failure of the treatments described above.^6^ Case series have shown success in the treatment of CLE, although there are no large studies to support this recommendation.^6^ It can be used by pregnant women with SLE, but its risk-benefit ratio must be weighed.^6^ The recommended dose is 1–3 mg/kg/day. Adverse effects are gastrointestinal, opportunistic infections, and cytopenias.^58^

### Thalidomide

Thalidomide is a drug used as rescue therapy in severe, refractory cases with a high risk of scarring.^66^ It works by inhibiting TNF-α synthesis, angiogenesis, and UVR-induced keratinocyte apoptosis, reducing IFN-γ production and phagocytosis by polymorphonuclear cells.^55^, ^69^ The response rate is greater than 90% in different subtypes of CLE, the highest among all available treatments,^6^, ^57^, ^70^ albeit there is high risk of recurrence, of up to 70%, after discontinuing the medication, especially in DLE. 6, 56, 70 The recommended starting dose is 100 mg/day, which should be reduced once clinical response is achieved.^61^

The high frequency of adverse events may limit its use, affecting 24% of patients (16% with peripheral neuropathy and 2% with thromboembolic events).^56^, ^70^ Polyneuropathy is classically symmetrical, painful, and affects hands and feet. It is usually accompanied by sensory loss and the preservation of muscle strength.^69^ An electroneuromyography (EMG) should be performed at baseline and every six months as control.^69^ Other side effects are sedation, orthostatic hypotension, maculopapular rash, constipation, and dry mouth.^67^, ^69^

Teratogenicity is one of the most feared side effects related to thalidomide use.^69^ Its use in women of childbearing age must be an exception, and only after the failure of all available treatments. In these cases, the use of two contraceptive methods is recommended, one highly effective and the other, a barrier one. A pregnancy test should be performed 24 hours before starting treatment, repeated weekly in the first month, and every two to four weeks thereafter.^69^

Acetylsalicylic acid, in low doses, can be combined with thalidomide in patients with high cardiovascular risk or the presence of antiphospholipid antibodies.^60^, ^69^

Lenalidomide, a thalidomide derivative, has a better safety profile regarding the risk of neuropathy, but there are still little data in the literature on its use in CLE patients.^6^, ^61^ Some authors contraindicate its use in CLE due to the risk of inducing SLE.^61^

### Systemic corticosteroids

Systemic corticosteroids can be used at the beginning of the treatment of aggressive and disseminated forms of CLE, until other medications start their therapeutic action. They should be reduced and discontinued as soon as possible.^1^, ^6^ They show a higher response rate in ACLE, likely due to their association with SLE.^61^ The usual dose is 0.5 to 1 mg/kg/day of prednisone and should be reduced as soon as possible, to reach daily doses of less than 7.5 mg. Long-term therapy with systemic corticosteroids is not indicated in CLE.^60^, ^61^

### Other treatments

Clofazimine has antimicrobial, anti-inflammatory, and immunosuppressive properties.^59^ The dose is 100 to 200 mg/day and can be used as an adjunctive treatment.^55^ The main side effects are brownish-gray hyperpigmentation, cutaneous xerosis, nausea, and vomiting.^55^ Fumaric acid esters have been successfully used in DLE, but data are still limited in the literature.^6^, ^57^

The use of antiplatelet agents is recommended in patients with livedo racemosa, malignant atrophic papulosis-like lesions (Degos disease), ulceration, thrombophlebitis, and anetoderma.^45^

The use of pulsed dye-laser is described as treatment for scarring. However, given the risk of photosensitivity, its use is not recommended in the presence of active skin lesions.^6^, ^7^, ^61^

Cyclosporine, cyclophosphamide, and intravenous immunoglobulin are not indicated in the treatment of CLE without systemic involvement.^1^, ^61^

### Target therapies

Advances in the understanding of the pathogenesis, especially of the activation pathways of the innate and adaptive immune systems, has opened a new field of research for a new generation of drugs, the so-called immunobiologicals.^56^ The main therapeutic targets are the activation pathways of B cells, T cells and pDC, in addition to pro-inflammatory cytokines, their receptors and intracellular signaling pathways, such as IL-6, IL-12, IL-23, IFN and JAK/STAT.^1^, ^14^, ^60^

**B-cell targeting**: belimumab is a monoclonal antibody against the B-cell activating factor (BlyS) approved by the FDA for use in SLE. The original studies did not include a specific analysis of the outcome of skin lesions, although later analyses have shown an improvement in the skin condition. Its efficacy in CLE is under investigation in phase III studies.^1^ Three observational studies using rituximab, an anti-CD20 monoclonal antibody, for the mucocutaneous manifestations of LE, showed response rates ranging from 35% to 76%. A more favorable response was seen in ACLE; however, with no evidence of a beneficial effect in the subacute and chronic subtypes of CLE.^57^

**Interferon pathway targeting**: Attempts at specific inhibition of IFN have not shown satisfactory results in clinical trials, probably because of the high redundancy between different types of IFN.^1^ The IFN receptor blockade shows a more promising prospect. Anifrolumab, a monoclonal antibody against type I IFN receptor, reduced skin lesion activity scores in patients with SLE in a phase IIb clinical trial.^1^

**JAK/STAT pathway targeting**: This pathway is important for the IFN upregulation. The first generation of inhibitors – baricitinib and ruxolitinib – showed to be effective in a small number of patients with perniotic LE. Second-generation inhibitors are being tested in clinical trials.^14^

## Final considerations

CLE is a multifactorial autoimmune disease, resulting from the interaction of environmental, genetic, and immunological factors, which presents varied dermatological manifestations. The identification of the clinical subtype is important for diagnostic approach, therapeutic decision, and determining the prognosis, both in exclusively cutaneous disease and in the context of SLE.

Diagnostic criteria for defining the different subtypes of CLE are still incipient. More assertive criteria are expected, which may be incorporated into clinical practice and therapeutic trials in the future, helping to assess the cutaneous manifestations of LE.

Photoprotection, topical corticosteroids and antimalarials are still the first lines of treatment for CLE. Alternative medications for systemic use include methotrexate, oral retinoids, dapsone, and thalidomide, among others. With advances in knowledge of disease pathogenesis, new therapeutic strategies have been developed, targeting the different immune activation pathways that have been identified.

## Financial support

None declared.

## Authors' contributions

Everton Carlos Siviero do Vale: Design and planning of the study; critical review of the literature; drafting and editing of the manuscript; critical review of the manuscript; approval of the final version of the manuscript.

Lucas Campos Garcia: Critical review of the literature; drafting and editing of the manuscript; approval of the final version of the manuscript.

## Conflicts of interest

None declared.

## References

[1] Wenzel J. (2019). Cutaneous lupus erythematosus: new insights into pathogenesis and therapeutic strategies. Nat Rev Rheumatol..

[2] Cooper E.E., Pisano C.E., Shapiro S.C. (2021). Cutaneous manifestations of “lupus”: systemic lupus erythematosus and beyond. Int J Rheumatol..

[3] Filotico R., Mastrandrea V. (2018). Cutaneous lupus erythematosus: clinico-pathologic correlation. G Ital Dermatol Venereol..

[4] Petty A.J., Floyd L., Henderson C., Nicholas M.W. (2020). Cutaneous lupus erythematosus: progress and challenges. Curr Allergy Asthma Rep..

[5] Jarrett P., Werth V.P. (2019). A review of cutaneous lupus erythematosus: improving outcomes with a multidisciplinary approach. J Multidiscip Healthc..

[6] Blake S.C., Daniel B.S. (2019). Cutaneous lupus erythematosus: a review of the literature. Int J Womens Dermatol..

[7] Batalla A., García-Doval I., Peón G., de la Torre C. (2013). A quality-of-life study of cutaneous lupus erythematosus. Actas Dermosifiliogr..

[8] Petri M., Orbai A.M., Alarcón G.S., Gordon C., Merrill J.T., Fortin P.R. (2012). Derivation and validation of the Systemic Lupus International Collaborating Clinics classification criteria for systemic lupus erythematosus. Arthritis Rheum..

[9] Aringer M., Costenbader K., Daikh D., Brinks R., Mosca M., Ramsey-Goldman R. (2019). 2019 European League Against Rheumatism/American College of Rheumatology classification criteria for systemic lupus erythematosus. Arthritis Rheumatol..

[10] Tan B.C.H., Tang I., Bonin J., Koelmeyer R., Hoi A. (2022). The performance of different classification criteria for systemic lupus erythematosus in a real-world rheumatology department. Rheumatology (Oxford)..

[11] Ribero S., Sciascia S., Borradori L., Lipsker D. (2017). The cutaneous spectrum of lupus erythematosus. Clin Rev Allergy Immunol..

[12] Hejazi E.Z., Werth V.P. (2016). Cutaneous lupus erythematosus: an update on pathogenesis, diagnosis and treatment. Am J Clin Dermatol..

[13] Elman S.A., Joyce C., Braudis K., Chong B.F., Fernandez A.P., Furukawa F. (2020). Creation and validation of classification criteria for discoid lupus erythematosus. JAMA Dermatol..

[14] Little A.J., Vesely M.D. (2020). Cutaneous lupus erythematosus: current and future pathogenesis-directed therapies. Yale J Biol Med..

[15] Oke V., Wahren-Herlenius M. (2013). Cutaneous lupus erythematosus: clinical aspects and molecular pathogenesis. J Intern Med..

[16] Li Q., Wu H., Zhou S., Zhao M., Lu Q. (2020). An update on the pathogenesis of skin damage in lupus. Curr Rheumatol Rep..

[17] Garelli C.J., Refat M.A., Nanaware P.P., Ramirez-Ortiz Z.G., Rashighi M., Richmond J.M. (2020). Current insights in cutaneous lupus erythematosus immunopathogenesis. Front Immunol..

[18] Hile G.A., Kahlenberg J.M. (2021). Immunopathogenesis of skin injury in systemic lupus erythematosus. Curr Opin Rheumatol..

[19] Li Q., Wu H., Liao W., Zhao M., Chan V., Li L. (2018). A comprehensive review of immune-mediated dermatopathology in systemic lupus erythematosus. J Autoimmun..

[20] Kudsi M., Nahas L.D., Alsawah R., Hamsho A., Omar A. (2021). The prevalence of oral mucosal lesions and related factors in systemic lupus erythematosus patients. Arthritis Res Ther..

[21] Roberts E.J., Melchionda V., Saldanha G., Shaffu S., Royle J., Harman K.E. (2021). Toxic epidermal necrolysis-like lupus. Clin Exp Dermatol..

[22] Nico M.M., Vilela M.A., Rivitti E.A., Lourenço S.V. (2008). Oral lesions in lupus erythematosus: correlation with cutaneous lesions. Eur J Dermatol..

[23] Borucki R., Werth V.P. (2020). Cutaneous lupus erythematosus induced by drugs - novel insights. Expert Rev Clin Pharmacol..

[24] He Y., Sawalha A.H. (2018). Drug-induced lupus erythematosus: an update on drugs and mechanisms. Curr Opin Rheumatol..

[25] Michaelis T.C., Sontheimer R.D., Lowe G.C. (2017). An update in drug-induced subacute cutaneous lupus erythematosus. Dermatol Online J..

[26] Bataille P., Chasset F., Monfort J.B., De Risi-Pugliese T., Soria A., Francès C. (2021). Cutaneous drug-induced lupus erythematosus: clinical and immunological characteristics and update on new associated drugs. Ann Dermatol Venereol..

[27] Szczęch J., Samotij D., Werth V.P., Reich A. (2017). Trigger factors of cutaneous lupus erythematosus: a review of current literature. Lupus..

[28] Antiga E., Caproni M., Bonciani D., Bonciolini V., Fabbri P. (2012). The last word on the so-called’ Rowell’s syndrome’?. Lupus..

[29] Derdulska J.M., Rudnicka L., Szykut-Badaczewska A., Mehrholz D., Nowicki R.J., Barańska-Rybak W. (2021). Neonatal lupus erythematosus - practical guidelines. J Perinat Med..

[30] Kus K.J.B., LaChance A.H., Vleugels R.A. (2021). Recognition and management of cutaneous connective tissue diseases. Med Clin North Am..

[31] Mirali S., Mufti A., Lansang R.P., Sachdeva M., Yeung J. (2021). Development of chronic cutaneous lupus erythematosus during biologic therapy: a systematic review. J Am Acad Dermatol..

[32] Menzies S., O’Shea F., Galvin S., Wynne B. (2018). Oral manifestations of lupus. Ir J Med Sci..

[33] Lospinoso D.J., Fernelius C., Edhegard K.D., Finger D.R., Arora N.S. (2013). Lupus erythematosus/lichen planus overlap syndrome: successful treatment with acitretin. Lupus..

[34] Schmitz S., Vatanchi M., Alapati U. (2018). Seven-year itch: a perplexing case of lichen planus-lupus erythematosus overlap syndrome. Dermatol Online J..

[35] Canu D., Viallard J.F., Lazaro E., Doutre M.S. (2021). Association of chilblain lupus and anti-Ro/SSA and anti-La/SSB antibodies: a study of 30 cases. Int J Dermatol..

[36] Dubey S., Joshi N., Stevenson O., Gordon C., Reynolds J.A. (2022). Chilblains in immune mediated inflammatory diseases: a review. Rheumatology (Oxford)..

[37] Cozzani E., Herzum A., Burlando M., Parodi A. (2020). Comedonal variant of chronic cutaneous lupus erythematosus causing mutilation of the earlobe. JAAD Case Rep..

[38] Chessé C., Fernández-Tapia M.J., Borzotta F. (2021). Comedonic lupus: an unusual presentation of cutaneous lupus. Actas Dermosifiliogr (Engl Ed)..

[39] Garcia L.C., Morato I.B., Melo R.F.Q., Vale E.C.S. (2023). Comedogenic lupus: a rare variant of chronic cutaneous lupus erythematosus - case series. An Bras Dermatol..

[40] Patsinakidis N., Kautz O., Gibbs B.F., Raap U. (2019). Lupus erythematosus tumidus: clinical perspectives. Clin Cosmet Investig Dermatol..

[41] Schmitt V., Meuth A.M., Amler S., Kuehn E., Haust M., Messer G. (2010). Lupus erythematosus tumidus is a separate subtype of cutaneous lupus erythematosus. Br J Dermatol..

[42] Aoki V., Vale E.C.S., Aoki V., Maruta C.W., Santi C.G. (2016). Dermatoses bolhosas autoimunes.

[43] de Risi-Pugliese T., Cohen-Aubart F., Haroche J., Moguelet P., Grootenboer-Mignot S., Mathian A. (2018). Clinical, histological, immunological presentations and outcomes of bullous systemic lupus erythematosus: 10 new cases and a literature review of 118 cases. Semin Arthritis Rheum..

[44] Rutnin S., Chanprapaph K. (2019). Vesiculobullous diseases in relation to lupus erythematosus. Clin Cosmet Investig Dermatol..

[45] Lenormand C., Lipsker D. (2021). Lupus erythematosus: significance of dermatologic findings. Ann Dermatol Venereol..

[46] Sampaio A.L., Bressan A.L., Vasconcelos B.N., Gripp A.C. (2021). Skin manifestations associated with systemic diseases - Part I. An Bras Dermatol..

[47] Bitar C., Menge T.D., Chan M.P. (2022). Cutaneous manifestations of lupus erythematosus: a practical clinicopathological review for pathologists. Histopathology..

[48] Kuhn A., Landmann A. (2014). The classification and diagnosis of cutaneous lupus erythematosus. J Autoimmun..

[49] Baltaci M., Fritsch P. (2009). Histologic features of cutaneous lupus erythematosus. Autoimmun Rev..

[50] Reimann J.D.R., Moynihan S.P., Horn T.D. (2021). Assessment of clinical and laboratory use of the cutaneous direct immunofluorescence assay. JAMA Dermatol..

[51] Didier K., Bolko L., Giusti D., Toquet S., Robbins A., Antonicelli F. (2018). Autoantibodies associated with connective tissue diseases: what meaning for clinicians?. Front Immunol..

[52] Zhou W., Wu H., Zhao M., Lu Q. (2020). New insights into the progression from cutaneous lupus to systemic lupus erythematosus. Expert Rev Clin Immunol..

[53] Chakka S., Krain R.L., Concha J.S.S., Chong B.F., Merola J.F., Werth V.P. (2021). The CLASI, a validated tool for the evaluation of skin disease in lupus erythematosus: a narrative review. Ann Transl Med..

[54] Mack E., Exton L.S., Mohd-Mustapa M.F., McCourt C., O’Kane D. (2021). Use of the Cutaneous Lupus Disease Area and Severity Index as an outcome measure in clinical trials: a descriptive study. Clin Exp Dermatol..

[55] Nutan F., Ortega-Loayza A.G. (2017). Cutaneous lupus: a brief review of old and new medical therapeutic options. J Investig Dermatol Symp Proc..

[56] Shi H., Gudjonsson J.E., Kahlenberg J.M. (2020). Treatment of cutaneous lupus erythematosus: current approaches and future strategies. Curr Opin Rheumatol..

[57] Fairley J.L., Oon S., Saracino A.M., Nikpour M. (2020). Management of cutaneous manifestations of lupus erythematosus: a systematic review. Semin Arthritis Rheum..

[58] Yan D., Borucki R., Sontheimer R.D., Werth V.P. (2020). Candidate drug replacements for quinacrine in cutaneous lupus erythematosus. Lupus Sci Med..

[59] Hannon C.W., McCourt C., Lima H.C., Chen S., Bennett C. (2021). Interventions for cutaneous disease in systemic lupus erythematosus. Cochrane Database Syst Rev..

[60] Chasset F., Francès C. (2019). Current concepts and future approaches in the treatment of cutaneous lupus erythematosus: a comprehensive review. Drugs..

[61] Kuhn A., Aberer E., Bata-Csörgő Z., Caproni M., Dreher A., Frances C. (2017). S2k guideline for treatment of cutaneous lupus erythematosus – guided by the European Dermatology Forum (EDF) in cooperation with the European Academy of Dermatology and Venereology (EADV). J Eur Acad Dermatol Venereol..

[62] Joseph A.K., Abbas L.F., Chong B.F. (2021). Treatments for disease damage in cutaneous lupus erythematosus: a narrative review. Dermatol Ther..

[63] de Sire A. (2021). Which interventions are effective for cutaneous disease in systemic lupus erythematosus? A Cochrane Review summary with commentary. Int J Rheum Dis..

[64] Chasset F., Bouaziz J.D., Costedoat-Chalumeau N., Francès C., Arnaud L. (2017). Efficacy and comparison of antimalarials in cutaneous lupus erythematosus subtypes: a systematic review and meta-analysis. Br J Dermatol..

[65] Reis Neto E.T., Kakehasi A.M., Pinheiro M.M., Ferreira G.A., Marques C.D.L., Mota L.M.H. (2020). Revisiting hydroxychloroquine and chloroquine for patients with chronic immunity-mediated inflammatory rheumatic diseases. Adv Rheumatol..

[66] Fanouriakis A., Kostopoulou M., Alunno A., Aringer M., Bajema I., Boletis J.N. (2019). 2019 update of the EULAR recommendations for the management of systemic lupus erythematosus. Ann Rheum Dis..

[67] Moura Filho J.P., Peixoto R.L., Martins L.G., Melo S.D., Carvalho L.L., Pereira A.K. (2014). Lupus erythematosus: considerations about clinical, cutaneous and therapeutic aspects. An Bras Dermatol..

[68] Klebes M., Wutte N., Aberer E. (2016). Dapsone as second-line treatment for cutaneous lupus erythematosus? A retrospective analysis of 34 patients and a review of the literature. Dermatology..

[69] Yuki E.F.N., Silva C.A., Aikawa N.E., Romiti R., Heise C.O., Bonfa E. (2021). Thalidomide and lenalidomide for refractory systemic/cutaneous lupus erythematosus treatment: a narrative review of literature for clinical practice. J Clin Rheumatol..

[70] Chasset F., Tounsi T., Cesbron E., Barbaud A., Francès C., Arnaud L. (2018). Efficacy and tolerance profile of thalidomide in cutaneous lupus erythematosus: a systematic review and meta-analysis. J Am Acad Dermatol..

